# Kv1.3 inhibition attenuates neuroinflammation through disruption of microglial calcium signaling

**DOI:** 10.1080/19336950.2020.1853943

**Published:** 2020-12-28

**Authors:** Alla F. Fomina, Hai M. Nguyen, Heike Wulff

**Affiliations:** a Department of Physiology and Membrane Biology, University of California, Davis, CA, USA; b Department of Pharmacology, University of California, Davis, CA, USA

**Keywords:** Microglia activation, Kv1.3, store-operated calcium influx, voltage-gated potassium channel, depolarization, neuroinflammation

## Abstract

In the last 5 years inhibitors of the potassium channel K_V_1.3 have been shown to reduce neuroinflammation in rodent models of ischemic stroke, Alzheimer’s disease, Parkinson’s disease and traumatic brain injury. At the systemic level these beneficial actions are mediated by a reduction in microglia activation and a suppression of pro-inflammatory cytokine and nitric oxide production. However, the molecular mechanisms for the suppressive action of K_V_1.3 blockers on pro-inflammatory microglia functions was not known until our group recently demonstrated that K_V_1.3 channels not only regulate membrane potential, as would be expected of a voltage-gated potassium channel, but also play a crucial role in enabling microglia to resist depolarizations produced by the danger signal ATP thus regulating calcium influx through P2X4 receptors. We here review the role of K_V_1.3 in microglial signaling and show that, similarly to their role in T cells, K_V_1.3 channels also regulated store-operated calcium influx in microglia.

## Introduction

The voltage-gated potassium channel K_V_1.3 was originally described in T cells [1] and subsequently pursued as a target for treating T-cell mediated autoimmune diseases such as multiple sclerosis, rheumatoid arthritis and psoriasis [2–4]. More recently K_V_1.3 has emerged as an attractive pharmacological target for reducing so-called “neuroinflammation” by modulating microglia activation. In both mouse and rat models of ischemic stroke K_V_1.3 blockers have been shown by us and others to reduce infarct area and improve neurological deficit [5,6]. In transgenic mouse models of Alzheimer’s disease K_V_1.3 blockade decreased cerebral amyloid load, enhanced hippocampal neuronal plasticity, and improved behavioral deficits [7]. Similarly, in multiple animal models of Parkinson’s disease administration of the small molecule K_V_1.3 blocker PAP-1 inhibited degeneration of dopaminergic neurons and improved behavioral outcomes [8]. K_V_1.3 inhibition or genetic deletion has further been shown to ameliorate white matter pathology after traumatic brain injury [9], postoperative cognitive decline [10], and radiation induced brain injury [11]. In all these animal models of neurological disease increased K_V_1.3 expression was observed on pathology-associated microglia and treatment with K_V_1.3 blockers resulted in reduced microglia activation and reduced inflammatory cytokine levels in the brains of the animals.

The disease-associated increased K_V_1.3 expression seems to translate to humans since strong K_V_1.3 expression has also been found in postmortem human brains on microglia in infarcted areas following ischemic stroke [5,12], on microglia surrounding amyloid plaques in Alzheimer’s disease [7,13], and on microglia in the prefrontal cortex and substantia nigra in Parkinson’s disease [8]. Taken together, these encouraging findings in humans and animal models strongly suggest microglial K_V_1.3 channels as a therapeutic target for reducing detrimental inflammatory responses in the brain. But how do K_V_1.3 blockers work? How does blocking a voltage-gated potassium channel in microglia, and presumably also brain infiltrating monocyte derived macrophages reduce inflammatory cytokine production and inflammation mediated tissue damage?

### Effects of K_V_1.3 blockers on microglia function

Cultured neonatal mouse and rat microglia, as well as acutely isolated microglia from mouse brain or tissue printed microglia from rat brain have been shown to express K_V_1.3 currents by whole-cell patch-clamp that increase substantially following *in vitro* stimulation or *in vivo* intracerebroventricular injection with the TLR-4 ligand lipopolysaccharide (LPS) [14–18]. Stimulation with the gram-negative cell wall component LPS is widely used to induce a “classically” activated “M1-like” state of microglia simulating general inflammation like would be associated with a bacterial infection [19,20]. In the field of neuroimmunology LPS stimulated microglia should ideally be called M(LPS) microglia based on the stimulus used to induce polarization to avoid using the M1/M2 terminology, which is regarded as oversimplified [21], but nevertheless widely used because of its therapeutic attractiveness. Following stimulation with LPS increased K_V_1.3 expression can be observed at the mRNA level and at the protein level by Western blotting, immunohistochemistry, and as increased current density measured by whole-cell patch-clamp [15–18]. A similar, but slightly less intense increase in Kv1.3 expression occurs when cultured neonatal microglia are stimulated with oligomeric amyloid-β (AβO) or α-synuclein [7,8], two proteins that are misfolded and aggregated in Alzheimer’s or Parkinson’s disease, and which are often used to create an *in vitro* model of microglia responses associated with the respective disease.

In these types of *in vitro* systems K_V_1.3 inhibition or genetic deletion reduces the expression and production of multiple inflammatory mediators measured at 24- or 48-hours following microglia activation. About 20 years ago the laboratory of Lyanne Schlichter reported that K_V_1.3 inhibitors suppress phorbol ester stimulated respiratory burst [22] and NO production in cultured neonatal rat microglia, thus reducing microglia mediated neuronal killing in a transwell culture system [16]. In a more recent study from our own laboratory [17], which compared the effects of K_V_1.3 and K_Ca_3.1 blockers with the widely used microglia inhibitor minocycline on differentially polarized neonatal mouse microglia, we observed that Kv1.3 inhibition reduced expression and production of the pro-inflammatory cytokines IL-1β and TNF-α at 24 and 28 hours and prevented upregulation of the inflammation associated enzymes cyclooxygenase-2 (COX-2) and inducible nitric oxide synthase (iNOS). The later effect was probably responsible for the reduced NO production observed in microglia treated with K_V_1.3 inhibitors at 24 and 48 hours after LPS stimulation. Similar to the earlier work, we also observed that K_V_1.3 inhibitors prevent microglia mediated neuronal killing in organotypic hippocampal slice cultures 3 days after microglia were activated either through 1-hour of hypoxia/aglycemia to simulate ischemic stroke [5] or by treatment with oligomeric amyloid-β to simulate microglia activation in AD [7]. In addition to reducing AβO induced microglia activation, a phenomenon which can be clearly shown by staining microglia for CD11b or Iba-1 and evaluating fluorescence intensity, the K_V_1.3 inhibitor PAP-1 has also been found to prevent AβO-induced, microglia-mediated impairment of long-term potentiation (LPT) in hippocampal slices and to reduce AβO-induced production of IL-1β, TNF-α and NO in cultured mouse microglia [7], very similar to what had been found for K_V_1.3 inhibitors with LPS stimulated microglia [17,18]. In experiments where cultured neonatal mouse microglia from wild-type and K_V_1.3^−/-^ mice were stimulated with aggregate α-synuclein (αSyn_Agg_), which activates microglia through binding to the pattern recognition receptors TLR2 and CD36 [23], both pharmacological K_V_1.3 inhibition with PAP-1 and genetic deletion of the channel resulted in reduced IL-1β, IL-6, IL-12 and TNF-α production and prevented NLRP3 inflammasome protein upregulation at 24 hours after microglia stimulation [8]. Conversely, overexpression of K_V_1.3 in a microglial cell line increased αSyn_Agg_-induced expression or secretion of IL-1β, TNF-α, IL-6 and IL-12 at 24 hours after stimulation [8]

Various studies by other groups confirmed that K_V_1.3 blockers reduce pro-inflammatory microglia functions when using similar stimuli or stimuli associated with other inflammatory pathologies. For example, in cultured rat microglia activated with the HIV Tat protein or with glycoprotein 120 (gp120) to simulate HIV-1-associated neurocognitive disorder, K_V_1.3 inhibition or knockdown with siRNA was found to reduce IL-1β, TNF-α and NO production and suppressed neurotoxicity [24,25]. Using a model of ischemic stroke and oxygen/glucose deprivation of cultured microglia Ma *et al*. reported that K_V_1.3 inhibition shifted the microglial phenotype from a pro-inflammatory M1-like to an M2-like response and reduced NLRP3 inflammasome activation [5,6], similar to the above described studies in models of Parkinson’s disease [8].

Using freshly isolated CD11b^+^ cells, which consist of both CNS infiltrating macrophages and brain resident microglia from the brains of mice that had been systemically treated with LPS in the presence and absence of the K_V_1.3 blocking peptide ShK-223, Rangaraju *et al*. confirmed that K_V_1.3 inhibition reduces expression of inflammatory cytokines like IL-1β but showed that K_V_1.3 inhibition also reduced expression of TAP1 and prevented upregulation of EHD1 in CD11b^+^CD45^low^ microglia as measured by flow cytometry [26]. Both TAP1 and EDH1 are proteins that are involved in MHC class-I assembly and trafficking to the cell surface. The authors therefore next investigated the effect of ShK-223 treatment on MHC class-1 expression on acutely isolated microglia, and, based on reduced MHC expression, hypothesized that LPS-induced increases in the antigen presenting capacity of microglia are K_V_1.3-dependent processes, suggesting that K_V_1.3 inhibition could reduce antigen presentation to CD8^+^ cytotoxic T cells. In a subsequent weighted co-expression network analysis applied to microglial transcriptomic datasets from neuroinflammatory and neurodegenerative disease mouse models the same group identified K_V_1.3 as part of a pro-inflammatory gene signature that also included Tlr2, Ptgs2, Il12b, Il1b, CD44, NFκB, Stat1, and RelA [27]. K_V_1.3 blockade with ShK-223 could shift this pro-inflammatory signature to one with increased expression of anti-inflammatory genes in disease associated microglia. What is interesting in this context, is that Kv1.3 inhibitors have been reported in other studies to further increase the migration of cultured microglia stimulated with the anti-inflammatory cytokines IL-4 and IL-10 [28] and to not affect microglial phagocytosis of microbeads [26] or to even increase phagocytosis of amyloid-β [7].

Taken together, there is thus ample evidence that microglia express K_V_1.3 and that K_V_1.3 blockers suppress pro-inflammatory, neurotoxic microglia functions and might even shift microglia toward a more neuroprotective phenotype. We here mostly concentrated on summarizing data obtained with cultured microglial or slice cultures because in these reductionist systems there is no doubt that knockout, knockdown or pharmacological blockade exerts its effect directly through inhibition of microglial K_V_1.3 channels and not indirectly through affecting K_V_1.3 channels in T cells, B cells, dendritic cells or monocytes, which all express K_V_1.3 [29], and could thus contribute to the *in vivo* effects of K_V_1.3 blockers or to findings made with K_V_1.3^−/-^ mice. However, we would like to point out here that in addition to numerous studies showing K_V_1.3 expression on microglia in rodents and humans by immunohistochemistry, the Kettenmann laboratory and our own group have repeatedly shown that increased K_V_1.3 expression on activated microglia is not just a tissue culture phenomenon and that Kv1.3 currents can be recorded from microglia in acute slices [30,31] and from acutely isolated microglia from the brains of 5xFAD mice [7] or from the infarcted area after middle cerebral artery occlusion [5,12], a model for ischemic stroke.

### Signaling pathways affected by K_V_1.3 inhibition or modulating Kv1.3 expression

**Figure 1.**
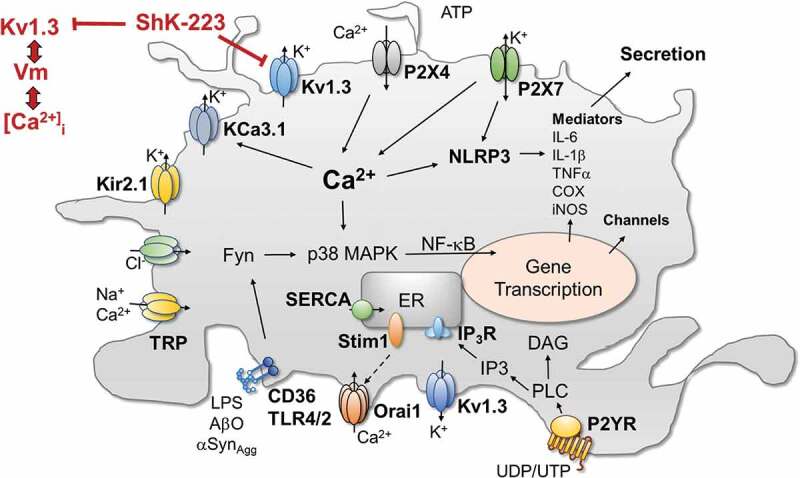
**Cartoon showing the involvement of K_V_1.3 and a selected number of other ion channels and receptors in microglia signaling**. Abbreviations: COX, cyclooxygenase; DAG, diacylglycerol; Fyn, Src-family of kinase; IL, interleukin; iNOS, inducible nitric oxide synthase; K_V_, voltage-gated K^+^ channel; K_ir_, inward-rectifier K^+^ channel; K_Ca_, calcium-activated K^+^ channel; IP_3_, ionositol-triphosphate; NF-κB, Nuclear factor kappa B; NLRP, nod-like receptor protein; Orai1, calcium release-activated calcium channel protein 1; P2YR, metabotropic purinergic receptor; P2X, ionotropic purinergic receptor; p38-MAPK, p38 mitogen-activated protein kinase; SERCA, sarco/endoplasmic reticulum Ca-ATPase; STIM, stromal interaction molecule; TLR, toll-like receptor; TNFα, tumor necrosis factor alpha; TRP, transient receptor potential channel

Studies probing into signaling pathways (Figure 1) that are affected by K_V_1.3 blockers to produce these beneficial reductions in pro-inflammatory microglia functions 24 or 48 hours later have rendered somewhat conflicting results, suggesting that the effects of K_V_1.3 blockers are dependent on the stimulus and maybe the species. While Fordyce *et al*. reported that K_V_1.3 blockers do not affect p38MAPK phosphorylation in LPS treated neonatal rat microglia [16], several other studies using mouse microglia have found that K_V_1.3 blockers strongly reduce p38MAPK phosphorylation as well as activation of NF-κB following stimulation with LPS, AβO, αSyn_Agg_ or HIV-1 glycoprotein 120 [7,8,24]. In the microglial cell line BV2 K_V_1.3 has also been shown to regulate S727 phosphorylation of STAT1 [26]. Interestingly, whether K_V_1.3 blockers inhibit NFAT translocation, one of the best studied effects of K_V_1.3 blockers in T cells [2,32,33] has not been investigated in microglia so far.

The increases in K_V_1.3 expression following stimulation of microglia with LPS, AβO or αSyn_Agg_ seem to involve many of the same signaling cascades that are affected by K_V_1.3 inhibition, and K_V_1.3 blockers typically prevent increases in Kv1.3 expression. The K_V_1.3 promoter contains multiple NF-κB and SP1 binding sites and inhibitors of NF-κB or p38MAPK independently reduce αSyn_Agg_ stimulated Kv1.3 up-regulation in microglia [8], demonstrating that both the p38MAPK and the classical NF-κB pathways mediate the transcriptional regulation of K_V_1.3 (Figure 1). The Src family kinase Fyn works upstream of the NF-κB transcription factor by regulating the nuclear translocation of its component p65 [34] and by phosphorylating the PKC isoform PKCδ. Sarkar *et al*. reported that Fyn knockout reduces both LPS and αSyn_Agg_ induced increases in K_V_1.3 current density in cultured microglia, an effect that can be mimicked by treatment of microglia with the Fyn inhibitor saracatinib or by deficiency for PKCδ, suggesting that the Fyn/PKCδ kinase signaling cascade is involved in mediating K_V_1.3 upregulation in response to inflammatory stimuli [8]. As demonstrated in a Duolink proximity ligation assay, Fyn is directly interacting with Kv1.3 and is phosphorylating the channel at Y135 to increase its activity.

### Kv1.3 regulates membrane potential and resists depolarization

Despite the above described well documented ameliorative effects of K_V_1.3 blockers on neuroinflammation *in vivo* and on microglial signaling cascades and inflammatory mediator expression and secretion *in vitro*, the fundamental molecular mechanisms of how K_V_1.3 blockers affect microglial functions was long unclear. Many investigators, our own laboratory included, simply assumed that K_V_1.3 was serving the same function in microglia that it was fulfilling in T cells, namely regulating membrane potential and calcium influx [29,32,33]. In T cells, K_V_1.3 inhibition leads to membrane depolarization and reduces calcium influx through calcium release-activated calcium channels (CRAC) following T cell receptor stimulation [33,35,36]. Calcium influx via CRAC channels is critically required for dephosphorylation and subsequent nuclear translocation of the calcium/calmodulin-dependent transcription factor NFAT [29], which triggers transcription of the cytokine IL-2 [29,33]. IL-2 is released from activated T cells upon synthesis and stimulates T cell clonal expansion in an autocrine and a paracrine manner. By inhibiting the counterbalancing potassium efflux that is necessary to sustain calcium influx through CRAC channels K_V_1.3 blockers disrupt T cells activation, prevent NFAT dephosphorylation and nuclear translocation [32] leading to reduced T cell proliferation and reduced inflammatory cytokine production and secretion at 48 hours [5,33,35,36]. However, it had never been tested in microglia, which unlike T cells also express the inward rectifier K_ir_2.1 [17,37,38] and the two-pore potassium channel THIK-1 (K_2P_13.1) [39], whether K_V_1.3 blockers would have the same molecular effects or if the role of K_V_1.3 was taken over by these other potassium channels. In a recent study our group accordingly addressed this question by first reexamining the fundamental significance of K_V_1.3 in maintaining resting membrane potential and resisting depolarization [40]. As expected, overexpression of K_V_1.3 in CHO cells lowered the typically very depolarized resting membrane potential of these cells from −20 mV to −50 mV, while K_V_1.3 inhibition with ShK-223 depolarized microglia, which inherently maintain a more hyperpolarized resting membrane potential, which in our hands averaged around −90 mV in undifferentiated microglia predominantly expressing K_ir_2.1 and around −65 mV in LPS stimulated microglia expressing higher K_V_1.3 and lower K_ir_2.1 current densities [40].

**Figure 2.**
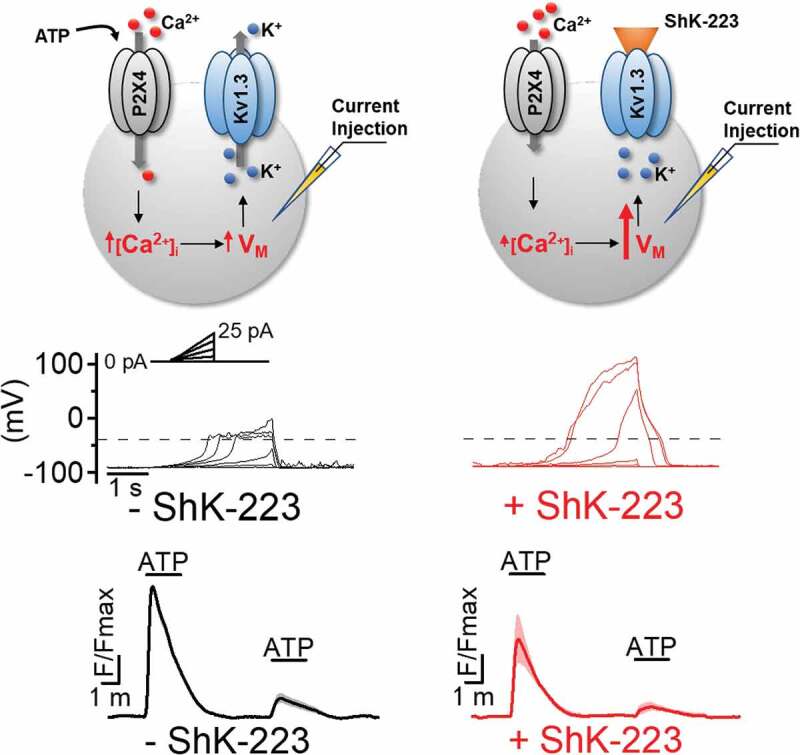
**K_V_1.3 regulates membrane potential and ATP-induced P2X4 receptor-mediated calcium entry**. K_V_1.3 enables microglia to resist depolarizing current injections in whole-cell patch-clamp experiments and to sustain larger ATP induced calcium signals in calcium imaging experiments. Both effects were inhibited or reduced by the K_V_1.3 blocking peptide ShK-223. For details see [40]

While K_V_1.3 thus certainly contributes to regulating resting membrane potential in microglia (Figure 2), we believe, its more important role, and the reason for why Kv1.3 expression increases in activated microglia, is the channel’s ability to offset extreme and prolonged membrane depolarization that potentially can negatively impact microglial health and physiology as revealed by current injection experiments. While current injections of 25 pA are not able to depolarize K_V_1.3 expressing microglia beyond −30 mV in the absence of blockers, microglia pretreated with K_V_1.3 blockers depolarize to potentials of +50 mV in response to the same 25 pA current injections [40], suggesting that K_V_1.3 is able to “clamp” membrane potential close to the channel’s activation threshold potential to prevent excessive membrane depolarizations (Figure 2). The situation in microglial physiology where this capacity is important is when microglia are exposed to extracellular ATP, a danger signal that can reach concentrations in the high micromolar range due to release of cytosolic ATP from damaged neurons or ATP-containing synaptic vesicles [41], which activate P2X receptor channels leading to intense depolarizations [42]. In our own hands, current clamp experiments performed on undifferentiated and LPS stimulated microglia showed that acute exposure to 100 μM ATP, which out of the P2X receptors expressed in microglia primarily activates P2X4, lead to more pronounced depolarizations in undifferentiated microglia, despite their more hyperpolarized resting membrane potential than in K_V_1.3^high^ LPS-stimulated microglia, which did not depolarize above −40 mV. K_V_1.3 blockers impaired this ability to resist ATP-induced depolarizations and microglia depolarized in their presence [40].

### K_V_1.3 modulates P2X4 receptor mediated calcium influx in microglia

Calcium imaging experiments revealed that this ability of K_V_1.3 channels to resist ATP induced P2X4-mediated depolarizations enhances Ca^2+^ entry into microglia through P2X4 receptors stimulated with 100 μM of ATP [40]. For example, we observed that the K_V_1.3 blockers ShK-223 and PAP-1 reduced ATP-induced Ca^2+^ influx through P2X4 receptors in LPS-differentiated microglia which express high K_V_1.3 current densities, much more strongly than in unstimulated microglia despite the fact that LPS stimulation downregulates P2X4 receptor expression in cultured microglia. In contrast, K_V_1.3 blockade had no effect on ATP-induced calcium transients in IL-4 stimulated microglia, which express very low levels of K_V_1.3. Interestingly, while LPS also increases K_V_1.3 and reduces P2X4 expression on microglia *in vivo*, both K_V_1.3 and P2X4 expression increase in parallel in the setting of ischemic stroke as shown by our laboratory on acutely isolated microglia from the infarcted brain hemisphere of mice subjected to ischemic stroke and by other groups on rodent and human microglia by immunohistochemistry or qPCR [5,12,43–46]. This suggests that following ischemic stroke K_V_1.3 might be regulating the more complex purinergic microglial calcium signaling initiated by the danger signal ATP more strongly than *in vitro*. ATP and other neuronal debris in the infarcted area of the brain activate microglia, which then continuously produce and release more inflammatory mediators within 24 to 72 hours and thus damage and kill more neurons in the penumbra leading to an inflammatory expansion of the infarct. The increased K_V_1.3 expression that is observed on microglia in the infarct border within 2–5 days [5] presumably enables activated microglia to sustain stronger calcium signals through the increased number of P2X4 receptors and thus to produce more inflammatory mediators, damaging more neurons and activating more microglia. This hypothesis would be in line with the finding that the K_V_1.3 blocker PAP-1 reduced microglia activation and brain levels of IL‐1*β* and IFN‐*γ*, leading to roughly 40% smaller infarct areas and improved neurological deficit scores in both mouse and rat models of ischemic stroke [5]. By depolarizing microglia Kv1.3 blockers reduce calcium influx through P2X4 receptors triggered by ATP released from damaged neurons and thus dampen further calcium-dependent activation of the NLRP3 inflammasome, NF-κB and other transcription factors leading to reduced inflammatory mediator production (Figure 1) and reduced pathology.

### What other calcium influx pathways in microglia are regulated by K_V_1.3?

While K_V_1.3 blockade clearly reduced ATP-induced calcium influx through P2X4 receptors [40] and thus constitutes an important regulator of this calcium influx pathway in microglia (Figure 2), this clearly is not the only K_V_1.3 or ATP dependent calcium signaling process in microglia. In addition to P2X receptor channels microglia also express metabotropic P2Y purinergic receptors which following activation by ATP or UTP can induce calcium release from internal stores [47] and activate store-operated Ca^2+^ entry (SOCE) following depletion of the relatively small intracellular calcium stores in microglia [38]. It therefore is certainly possible that additional SOCE contributed to the ATP induced Ca^2+^ signals we had imaged and that some of the K_V_1.3 blocker effects were mediated by additionally reducing SOCE. This is a very likely hypothesis, since the “classical” molecular mechanism for K_V_1.3 blockers in T cells is reduction of SOCE through CRAC channels following T cell receptor activation and subsequently reduced transcription factor activation and cytokine production and release [29,33,36,48]. Rat and mouse microglia have been repeatedly reported to exhibit currents with biophysical properties typical for CRAC currents [49,50] and to express the molecular components of CRAC channels, Orai and STIM [51,52]. There further is evidence that genetic deletion of Orai1, STIM1, and STIM2 reduces CRAC current and/or SOCE and inhibits migration and phagocytosis in cultured mouse microglia [52], while the small molecule CRAC channel blocker CM-EX-137 has recently been reported to decrease microglia activation and lesion size in a mouse model of traumatic brain injury [53].

### Blockade of K_V_1.3 channels attenuates store-operated calcium entry in LPS stimulated microglia

**Figure 3.**
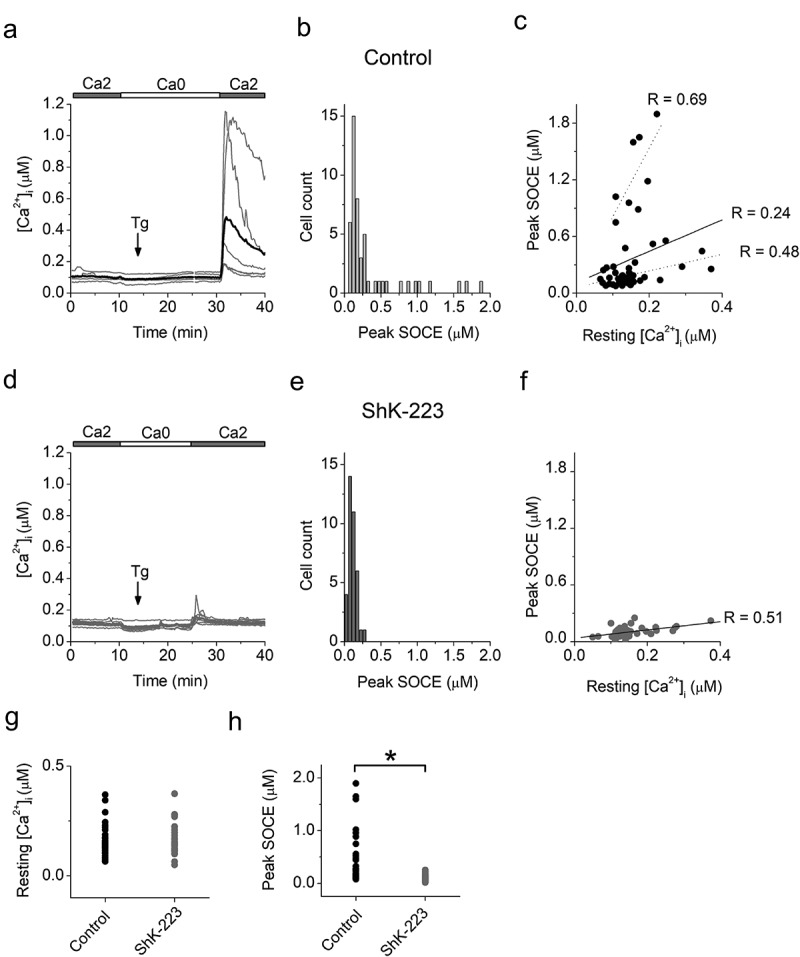
**The K_V_1.3 blocker ShK-223 reduces SOCE in LPS differentiated neonatal microglia**. Primary neonatal microglia cultures were prepared from 3 independent litters and stimulated with LPS as described in our publication investigating the effect of K_V_1.3 blockers on P2X4 mediated Ca^2+^ influx in microglia [40]. Loading microglia with Fura-2 was performed as previously described [56]. Fura-2 fluorescence signals evoked by 340- and 380-nm excitation (*F*_340_ and *F*_380_, respectively) were acquired every 12 s. Data acquisition was performed using MetaFluor version 7.0 software (Molecular Devices). Estimated [Ca^2+^]*_i_* was calculated from background-subtracted *F*_340_/*F*_380_ ratio values and Fura-2 calibration as previously described [57]. Differences between data sets were analyzed using one-way analysis of variance (one-way ANOVA) with Bonferroni correction (Origin 7 software)

As a follow-up experiment to our previous paper on the role of K_V_1.3 in regulating P2X4 mediated calcium influx, we here specifically asked the question if the K_V_1.3 blocker ShK-223 would also reduce SOCE in LPS differentiated microglia, the same stimulation condition, where we had observed the highest K_V_1.3 current densities and the largest effects of K_V_1.3 inhibition on ATP induced calcium signaling [40]. Using the ratiometric calcium indicator Fura-2 to track changes in the free intracellular calcium concentration ([Ca^2+^]_i_) we found that LPS differentiated microglia had a resting cytosolic calcium concentration [Ca^2+^]_i_ that varied between 60 nM and 370 nM, averaging 150 ± 8.8 nM (n = 50; Figure 3a,c,g), which is consistent with a previous report [54]. To elicit SOCE, intracellular calcium stores were first depleted with thapsigargin (Tg), a blocker of the endoplasmic reticulum Ca^2+^-ATPase, applied in a nominally Ca^2+^-free bath solution. Removal of extracellular calcium produced a roughly 25 nM decrease in [Ca^2+^]_i_ (Figure 3a) indicating the presence of a calcium influx at rest. Subsequent application of Tg (1 μM) elicited small amplitude (> 25 nM) asynchronous elevations in [Ca^2+^]_i_ due to passive calcium mobilization from stores. Re-addition of calcium into the bath solution elicited a rapid elevation in [Ca^2+^]_i_ above the resting levels caused by SOCE. The magnitude of the SOCE-induced [Ca^2+^]_i_ transients (“peak SOCE”) measured as the difference between [Ca^2+^]_i_ level in the Ca^2+^-free bath solution and maximal [Ca^2+^]_i_ elevation following calcium re-addition varied significantly among individual cells with peak SOCE distribution displaying a maximum at 120 nM and a tail up to 1800 nM (Figure 1b). This heterogeneity in the strength of SOCE is likely caused by variations in resting membrane potential, which provides the driving force for calcium entry during SOCE. The resting membrane potential of LPS-differentiated microglia has been found to vary between −90 mV and −40 mV in individual cells [40,55] and thus could account for the observed differences in SOCE.

(a and d), Changes in [Ca^2+^]_i_ recorded from LPS-stimulated microglia in the absence (a) or after preincubation with 100 nM ShK-223 (d). Shown are the data from one representative experiment with microglia from one neonatal culture. Grey traces are recordings from individual cells. The thick black trace and the thick red traces in (a) and (d), correspondingly, are averages of the traces from the individual cells. 2 mM Ca^2+^-containing (2Ca) and nominally Ca^2+^-free (0Ca) solutions were applied as indicated by horizontal bars; the timing of application of Tg (1 μM) is indicated with an arrow.(b and e), Distributions of the amplitudes of the [Ca^2+^]_i_ transients following readdiction of extracellular Ca^2+^ (peak SOCE) in untreated control cells (b), and cells preincubated with ShK-223 (e). Pooled data from 4 paired experiments from 3 independent microglia preparations from 3 litters. The total number of cells is 50 in B and 37 in (e).(c and f), Relationship between peak SOCE and resting [Ca^2+^]_i_ in untreated control cells (c) and cells preincubated with ShK-223 (f). Straight lines are linear regression fits of the whole set of data. The Pearson correlation coefficients (R), were 0.24 in (c) (n = 50) and 0.44 in (f) (n = 37), indicating weak and mild positive correlation, correspondingly. Deashed lines in (c) indicate linear regression fits of the data from subpopulations that include cells with amplitudes of peak SOCE above and below 600 nM. The Pearson correlation coefficients (R), were 0.69 (n = 8) for cells with peak SOCE > 600 nM and 0.48 (n = 42) for cells with peak SOCE < 600 nm, indicating strong and mild positive correlation, respectively. (c and f), pooled data from 4 experiments from 3 independent microglia preparations from 3 litters. n, number of cells.(g), Resting [Ca^2+^]_i_ in control cells (black dots, n = 50) and cells preincubated with ShK-223 (red dots, n = 37).(h), Peak SOCE in control cells (black dots, n = 50) and cells preincubated with ShK-223 (red dots, n = 37). Star indicates that the difference between groups is significant at 0.05 level (one-way ANOVA with Bonferroni correction, α = 0.0083). (g) and (h) show pooled data from 4 paired experiments on cells isolated from 3 independent microglia preparations from 3 litters. n, number of cells.

A previous study reported that elevation in resting [Ca^2+^]_i_ was associated with a reduced ability of external stimuli such as the P2Y receptor agonist UTP or complement factor 5a to elicit [Ca^2+^]_i_ transients in LPS-activated microglia [54]. Because in our study both resting [Ca^2+^]_i_ levels and peak SOCE values varied among individual cells, we explored whether variation in resting [Ca^2+^]_i_ may account for heterogeneity in peak SOCE values. Plotting peak SOCE values versus resting [Ca^2+^]_i_, revealed a positive correlation between peak SOCE and resting [Ca^2+^]_i_ (Figure 3c). When we split the data into two groups with a cutoff value of 600 nM for peak SOCE, the relationships between resting [Ca^2+^]_i_ and peak SOCE in each group were stronger than in the whole population, indicating the presence of at least two cell populations, which differ in both magnitude of SOCE and strength of correlation between resting [Ca^2+^]_i_ and peak SOCE. Thus, within the population of LPS-treated cells, an elevation in resting [Ca^2+^]_i_ is not causally linked to the reduced ability of activated microglia to respond to external stimuli with a [Ca ^2+^]_i_ transient, as was previously suggested by Hoffman *et al*. Our data suggest that a common influence, such as the level of membrane potential, which is controlled by a variety of channels, including K_V_1.3, K_ir_2.1 and THIK-1, may be responsible for the positive correlation between resting [Ca^2+^]_i_ and peak SOCE observed in our study. For example, overexpression or functional upregulation of hyperpolarizing channels could hyperpolarize the membrane and increase the driving force for both calcium entry via constitutively active calcium permeable channels such as metabolically activated transient receptor potential (TRP) channels and store-operated channels activated following store depletion such as CRAC channels.

Pre-incubation of cells with the K_V_1.3 inhibitor ShK-223 did not significantly affect resting [Ca^2+^]_i_ (Figure 3d,g), but eliminated peak SOCE responses larger than 300 nM and shifted the maximum of the distribution of peak SOCE values from 130 nM (Figure 3b, control) to 70 nM (Figure 3e, ShK-223). The difference between the values of peak SOCE in control cells and cells preincubated with ShK-223 was significant (Figure 3h). The relationship between resting [Ca^2+^]_i_ and peak SOCE still displayed a mild positive correlation (figure 3f). Taken together, our data indicate that similarly to T lymphocytes, blockade of K_V_1.3 channels significantly reduces SOCE in LPS differentiated microglia. The extent of the inhibition varied among cells with a greater effect on cells expressing large magnitude (> 300 nM) SOCE. The specific characteristics of the cells with high and low peak SOCE remain to be determined.

Under physiological conditions, SOCE can be triggered subsequently to store depletion following activation of G-protein coupled P2Y receptors and contributes to the combined [Ca^2+^]_i_ elevation during activation of P2Y and P2X receptors elicited by common purinergic agonists, such as ATP. The magnitude of the SOCE varies significantly among LPS differentiated cells, which may be a function of the unique combination of differential expression of store-operated channels and channels controlling membrane potential, such as K_V_1.3. Reduction in peak SOCE by ShK-223 indicates that K_V_1.3 channels are potent regulators of SOCE and uncover an additional mechanism by which K_V_1.3 blockers attenuate calcium signaling and subsequent functional responses (e.g. cytokine production) following calcium dependent transcription factor activation in microglia.

## Conclusions

While it is certainly challenging to sort out the contributions effects on cells of the peripheral immune system, particularly on memory T cells and monocyte derived macrophages, make to the beneficial effects of K_V_1.3 blockers on inflammation in the CNS, it is clear that K_V_1.3 plays important roles in regulating membrane potential, preventing depolarization and controlling calcium signaling events that subsequently lead to microglia activation and inflammatory mediator production. We would like to suggest that from a therapeutic perspective it is probably desirable to inhibit K_V_1.3 in multiple immune system cell types. However, it would be intellectually very satisfying and helpful for therapy development to clarify what the major cellular target of K_V_1.3 inhibitors is and whether therapeutics need to be brain penetrant in order to exert optimal ameliorating effects in CNS diseases. These questions should ideally be addressed both pharmacologically by testing brain penetrant small molecules side by side with peripheral restricted petidic K_V_1.3 inhibitors or anti-K_V_1.3 antibodies [58,59] in animal models of Alzheimer’s and Parkinson’s disease. It would further be highly informative to compare the effects of conditional K_V_1.3 deletion in microglia, monocyte derived macrophages and different T cell subsets to the effects of drug treatment to gain a more detailed understanding of the role of K_V_1.3 in neuroinflammation and the cellular target of K_V_1.3 inhibitors. However, we believe that at the cellular level the fundamental role of K_V_1.3 will always be what was first described in T cells, namely regulation of membrane potential and calcium influx and subsequent activation of calcium dependent transcription factors. What sometimes does not get stressed enough in this respect, is probably that K_V_1.3’s biophysical properties like its half-activation voltage and its relatively slow inactivation [60,61], perfectly “qualify” the channel to resist membrane depolarizations and thus enable K_V_1.3 to not only regulate SOCE, as was described 30 years ago in T cells, but also P2X receptor mediated calcium influx.
